# Potential Roles of the Renin-Angiotensin System in the Pathogenesis and Treatment of COVID-19

**DOI:** 10.1155/2020/7520746

**Published:** 2020-11-05

**Authors:** Ling Lu, Xiaomei Liu, Rong Jin, Renzheng Guan, Rongjun Lin, Zhenghai Qu

**Affiliations:** Department of Pediatrics, The Affiliated Hospital of Qingdao University, Qingdao 266003, China

## Abstract

The spread of pathogenic severe acute respiratory syndrome-coronavirus-2 (SARS-CoV-2) poses a global health emergency. Based on the symptomatic treatment and supporting therapy, prevention of complications is the major treatment option. Therefore, it is necessary to illustrate the potential mechanisms for the pathogenesis of COVID-19. Angiotensin-converting enzyme 2 (ACE2), the major receptor of SARS-CoV-2, is one of the major members of the renin-angiotensin system (RAS). In this review, we aimed to summarize the crucial roles of ACE2 in the pathogenesis of COVID-19, followed by illustrating potential treatment options relating to ACE2 and the RAS.

## 1. Introduction

Coronavirus disease 2019 (COVID-19) is a new type of acute respiratory syndrome that emerged in Wuhan City, Hubei Province, China, in December 2019 [1]. It has caused an outbreak of pneumonia as it is highly transmissible [2], rapidly spreading in a pandemic to more than 190 countries including the USA, India, and Brazil, which are severely affected. Until October 6, 2020, the total number of cases infected with COVID-19 reached more than 34 million, leading to a death toll of one million. This is a worldwide pandemic event that severely harms the life, safety, and public health.

The priority of COVID-19 management in clinical settings is to save human lives, especially those who are critically ill or extremely ill. It is a challenge to reduce the death toll. According to the COVID-19 guidelines proposed by the Chinese Medical Association, people were susceptible to COVID-19 with various clinical manifestations [3, 4]. Patients with mild or moderate conditions showed low-grade fever and slight fatigue without pneumonia, while those with severe conditions mainly presented respiratory failure and/or multiple organ failure characterized by refractory and severe hyoxemia [5, 6]. To date, most patients showed a satisfactory outcome and some cases developed critically ill conditions. Generally, the prognosis was usually poor in the aged population and those with chronic underlying diseases.

Despite the great strides that have been made in the knowledge of COVID-19, the mechanism of how the virus affects our body is still not clear. Particularly, little is known about the severe conditions among some critically ill patients. In this review, we tried to discover the individual variation of the symptoms after infection in terms of angiotensin-converting enzyme 2 (ACE2) and the renin-angiotensin system (RAS).

## 2. SARS-CoV-2 Entered the Cells through the ACE2

The virus responsible for the COVID-19 infection is a member of the beta coronavirus family, wrapped with an envelope. The particle is round or elliptical in profile, with a diameter of about 60-140 nm. Similar to the other known coronaviruses, OC43, HKU1, MERS-CoV, and SARS-CoV, SARS-CoV-2 was reported to be infectious to the human beings. It has been commonly acknowledged that the virus is likely derived from wild animals. The most probable hosts are bats, but the intermediate hosts are still not well defined [7]. The spread of an animal virus would basically rely on its binding with the corresponding receptors in human cells. To our best knowledge, the S protein of the coronavirus is responsible for binding with the receptor protein in the hosts, which facilitated the invasion of virus to the host cells. The spike (S) protein exists in the form of a tripolymer, which formed a typical envelope apophysis including an extracellular segment, a single-pass receptor anchoring segment, and an intracellular segment [8]. The extracellular segment was formed by S1 and S2 subunits. The S1 subunit consisted of the N-terminal domain (NTD) and the receptor-binding domain (RBD). Upon invasion into the host cells, S1 could bind with the receptors on the cell surface, while S2 could mediate the membrane fusion, which contributed to the virus entry to the host cells [9]. The spread of SARS-CoV-2 to human beings is mainly based on the same binding receptor that has been reported in SARS-CoV [10]. In a previous study, compared with the corresponding regions of SARS, the core components of the SARS-CoV-2 were similar except for sequence changes of four amino acids among the five crucial amino acids in the S protein receptor-binding domain [9]. The core components could interact with ACE2, which explained the possible mechanism of the pandemic spread of COVID-19 [9]. Zhou et al. [1] reported that SARS-CoV-2 could bind with the ACE2 in the human body, bat, palm civet, and pigs, while no infection was induced in animals without ACE2. Meanwhile, SARS-CoV-2 could not bind with the other receptors such as APN and DPP4 [1]. Furthermore, these studies indicated that ACE2 was the receptor used by SARS-CoV-2, which was similar to SARS-CoV.

Viral adsorption to the cell surface of the hosts was the initial step for the binding of virus to the receptors. The viral receptor could recognize the host protein on the cell surface, followed by binding with the protein. This was the major pacing factor for inducing infection. During the invasion by SARS-CoV-2, ACE2 showed crucial roles and served as the only key to open the cellular door. In a recent study, Yan et al. [10] revealed that ACE2 showed a dipolymeric structure, presenting two different conformations in an open or closed state. These two conformations contained the mutual recognition surface with the SARS-CoV-2. Besides, the binding capacity of the S protein of SARS-CoV-2 to the ACE2 was about 10-fold higher than that of SARS-CoV.

TMPRSS2 played important roles in the splicing and activation of the S protein during the binding of SARS-CoV-2 and ACE2 [11]. There are two conditions required for the S protein of coronavirus to enter into the target cells. Firstly, the subunit S1 of the S protein binds to the cell receptor so that the virus could attach to the surface of the target cell [9]. Secondly, the cell protease is required to lyse the S1/S2 and S2′ sites of the S protein to promote the fusion of virus and the cell membrane. It has been widely acknowledged that the S protein of SARS-CoV binds with ACE2 to enter into the cell and uses the cell serine protease TMPRSS2 to initiate the S protein reaction [12]. The availability of ACE2 is the key to the spread of SARS-CoV. The SARS-CoV S protein and SARS-CoV-2 S protein are 76% similar in amino acid composition [13]. Hoffmann et al. [14] indicated that SARS-2-S, like SARS-S, employed ACE2 and TMPRSS2 for host cell entry. Moreover, in a previous study, TMPRSS2 could splice the C-termini of ACE2 during the activation of the S protein, which enhanced the entry of the S protein into the host cells [15].

## 3. Function and Distribution of ACE2

**Figure 1:**
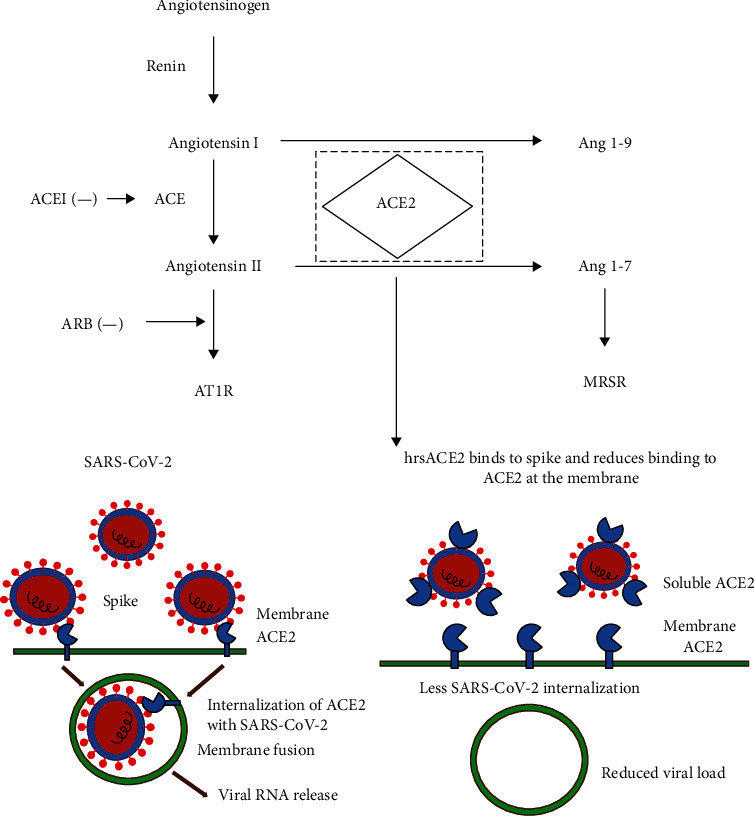
Prehypertensin could cleave into Ang I in the presence of rennin, which then transformed into Ang II. Ang II would bind with the AT1R and AT2R, which then contributed to the tissue fibrosis and inflammation. Ang II deactivated by ACE2 could generate Ang 1-7 that could bind with the Mas receptor, which was involved in the anti-inflammation and antifibrosis. Angiotensin-converting enzyme inhibitor (ACEI)/ARB could inhibit the ACEI/Ang II/AT1 axis and eliminate the Ang II formation and the stimulation of AT1, which then attenuated the inflammatory reactions. The S protein could bind with ACE2, and then, the SARS-COV-2-ACE2 entered the cells, followed by membrane fusion. Then, the virus was released to the host cells. The decline of ACE2 and RAS imbalance would lead to inflammatory reactions. The ectogenous supplementation of hrsACE2 could block the entry of SARS-CoV-2 to host cells. The ACE2 level was normal, which showed protective effects to the hosts.

As a type I transmembrane glycoprotein, ACE2 contains two domains [16]. ACE2 could catalyze the transmission of angiotensin I into Ang 1-9. Also, it could catalyze the transmission of angiotensin II (Ang II) to Ang 1-7 (Figure 1). Its major functions were as follows: (a) as a negative regulator of RAS, it is involved in the balancing of ACE functions, especially the vasodilatation that played important roles in the protection of the cardiovascular system and other organs [17]; (b) it served as an important receptor for HCoV-NL63, SARS-CoV, and SARS-CoV-2 [1, 10, 18]; and (c) it could bind with B0AT1 in the kidney and the intestinal tract, which assisted in the absorption of the amino acids [19]. ACE2 was widely distributed on the surface of alveolar epithelial cells (AEC) [20, 21], which was consistent with the fact that the lung was the main target organ of COVID-19. Enrichment analysis of gene ontology showed that ACE2-expressing AECII cells expressed high levels of multiple genes related to virus processing, including virus processing regulation genes, virus life cycle genes, and virus assembly and virus replication genes [22]. Thus, AECII cells expressing ACE2 promotes the replication of coronavirus in the lungs [23]. In addition to lung tissue, ACE2 is also expressed in the heart, renal tubules, lumen surfaces of the small intestine, blood vessels, and other tissues. The tissue distribution of ACE2 in other organs can clarify multiorgan dysfunction in some patients [24–26].

## 4. Roles of ACE2 and RAS in COVID-19 and ARDS

Acute respiratory distress syndrome (ARDS) is a very severe acute lung injury that affects about 1 million people worldwide each year and causes 30-50% of deaths [27, 28]. It could be induced by several conditions including aspiration, sepsis, acute pancreatitis, trauma, or pneumonia after being affected by SARS coronavirus, SARS-CoV-2, or avian and human influenza viruses. According to the previous description, ACE insertion and/or deletion was correlated with the severity of ARDS among humans [29, 30]. In rodent ARDS models [31], an ACE inhibitor prevented the development of ARDS or acute lung injury (ALI), which demonstrated that the RAS might be associated with the pathogenesis of ARDS or ALI.

Compared with the wild-type mice, ACE2-knockout mice showed severe ARDS and/or acute lung injury [32], accompanied by increased vascular permeability, pulmonary edema, and neutrophil infiltration. In addition, AT1 blocker or ACE gene deletion in the context of ACE2 knockout can save the severe phenotype of a single ACE2 mutant in acute lung injury [32]. Notably, symptoms of acute lung injury in wild-type and ACE2-knockout mice were improved by recombinant ACE2 protein, which can also significantly reduce respiratory failure of the septic ARDS model (pigs) and increase their oxygen saturation [33]. Based on it, ACE, Ang II, and AT1 receptors may play a role as lung injury promoters in animal models with acute lung injury; meanwhile, ACE2's negative regulation of Ang II level had a protective effect on lung injury animals [32]. In addition, ACE2 has been proven to play a protective role in chronic lung injury, pulmonary fibrosis, and pulmonary vasoconstriction in other lung injury models, for example, bleomycin-induced pulmonary fibrosis models and monocrotaline-induced pulmonary hypertension models [34, 35].

SARS-COV infection and the attack of the recombinant SARS spike protein could both induce significant downregulation of ACE2 expression in lung tissue [36]. Therefore, mice infected with SARS coronavirus or treated with the spike protein are similar to ACE2-knockout mice. Like ACE2 mutant mice, wild-type mice treated with the S protein showed more severe pathological manifestations in acute lung injury [36]. The ACE2/angiotensin (Ang) 1-7 axis is not taken as the classical RAS axis, which is the endogenous inverse regulatory axis of the ACE/Ang II axis [10, 37]. Based on this, downregulated expression of ACE2, the SARS receptor, can activate RAS, which contributes to the occurrence of severe acute lung injury of SARS [38].

There was indeed a high similarity between the SARS virus and SARS-CoV-2. Patients with SARS and COVID-19 all presented with fever and cough, which frequently resulted in lower respiratory tract disease with poor clinical outcomes [39]. Meanwhile, based on the pathological samples after biopsy puncture, Xu et al. [40] found obvious desquamation of pneumocytes and hyaline membrane formation in the right lung and pulmonary edema with hyaline membrane formation in the left lung tissue. The pathological features of COVID-19 were remarkably similar with those in SARS- and MERS-infected patients [41, 42]. Thus, we speculated that there might be changes in the ACE2 and RAS after SARS-CoV-2 infection. Monteil et al. [43] indicated that the S protein of SARS-CoV-2 could bind with ACE2 on the cellular membrane of the host cells, and then, the SARS-CoV-2-ACE2 complex entered the cells. After membrane fusion, the virus would release into host cells. The ACE2 on the cellular membrane of the host cells showed a decline, and RAS showed imbalance, which triggered the inflammatory reactions. Administration of human recombinant soluble ACE2 (hrsACE2) could block the entry of SARS-CoV-2 into human host cells. A high level of expression of ACE2 in human host cells showed protective effects. The most concerned point is that why the lung is the most susceptible organ. There are two reasons. The first is that the lung's superfacial area is quite large, so it is more vulnerable to the viruses. The other reason is that 83% of ACE2-expressing cells are type II alveolar epithelial cells (AECII) [44], which mainly exist in the lung, making the lung tissue a storage vault for viruses.

## 5. ACE2 or the Inflammatory System RAS May Serve as Treatment Regimens for COVID-19

As previously described, patients infected with COVID-19 showed clinical symptoms of ALI and ARDS. There were indeed similarities between COVID-19 and SARS and MERS. As COVID-19 shared the same receptor with SARS, more studies will be required to focus on the development of new treatment options based on the previous studies on SARS. Recently, more and more studies have focused on the roles of ACE2 and the inflammatory regulation in animals with ALI or ARDS. In an ARDS rat model induced by cigarette smoke inhalation, there was augmented expression of ACE and ACE2 in the lung tissues [25]. In addition, there was a positive correlation between ALI severity and age-dependent reduction of the ACE2/ACE ratio [26]. In a LPS-induced ALI rat model, losartan could attenuate the inflammatory response and pulmonary injury [45]. In a recent phase II trial on GSK2586881 that served as a recombinant form of human ACE2, there was good tolerance for GSK2586881 among the ARDS patients. Moreover, it could trigger a decline of Ang II levels and an elevation of Ang 1–7 and surfactant protein D levels [11]. In rats with hydrochloric acid-induced ALI, subcutaneous infusion of Ang 1–7 contributed to the attenuation of pulmonary cell infiltration and fibrosis, which indicated that Ang 1-7 showed protective roles against ALI [46]. Besides, Ang 1–7 could attenuate ventilator-induced and acid aspiration-induced ALI in mice, as well as oleic acid model of ALI in rats [47]. In a recent study on an H5N1 virus-induced ALI model, there was amplified expression of miR-200c-3p triggered by the NF-*κ*B signaling pathway, in combination with a decline of ACE2 and an increase in Ang II. Moreover, inhibition of miR-200c-3p serving as a microRNA that targeted the 30-untranslated region of ACE2 was reported to show protective effects on lung injury and ARDS induced by H5N1 virus in mice [48]. After taking this information into consideration, we speculated that blockade of AT1R or activation of the ACE2/Ang 1–7/Mas receptor pathway may potentially be promising candidates for therapeutic strategies against ALI [24].

Hoffmann et al. [14] pointed out that the SARS-CoV-2 S protein bound with the ACE2 receptor and entered into cells, and the cell serine protease TMPRSS2 was used to initiate the S protein reaction. Serine protease inhibitors can be used to block the invasion of the virus clinically and possibly are one of the alternative ways to treat SARS-CoV [14]. SARS-CoV-2 entered into the respiratory tract by binding its surface S protein to ACE2, the membrane protein of type II alveolar epithelial cells. The S protein-ACE2 complex was internalized into cells through endocytosis, resulting in reduced or complete inactivation of the enzymatic functions of ACE2 existing in alveolar cells (Figure 1). This further reduced the degradation of proinflammatory angiotensin II and reduced the concentration of its physiological antagonist angiotensin 1-7, thereby increasing the tissue concentration of proinflammatory angiotensin II. Abundant Ang II in the lung interstitium promoted apoptosis, enabled inflammatory response, freed proinflammatory cytokines, built self-cascade response, and finally resulted in ARDS. Lately, Gurwitz [48] recommended the initial use of losartan and telmisartan as an alternative treatment in COVID-19 patients before evolving into ARDS. Zhang et al. [49] found that among patients with hypertension hospitalized with COVID-19, inpatient treatment with ACEI/ARB was associated with lower risk of all-cause mortality compared with ACEI/ARB nonusers. In a previous study [50], the authors proposed telmisartan as an alternative drug based on its pharmacokinetic and pharmacodynamic properties and designed an open-label randomized phase II clinical trial to evaluate telmisartan in patients with COVID-19 (NCT04355936). Besides, it confirmed that the underlying cardiovascular disease was associated with an increased risk of in-hospital death among patients hospitalized with COVID-19 [51]. Their results did not confirm a potential harmful association between ACE inhibitors and ARBs on in-hospital death in the clinical context. Reynolds et al. found no substantial increase in the likelihood of a positive test for COVID-19 or in the risk of severe COVID-19 among patients that were positive in association with five common classes of antihypertensive medications [52]. On this basis, patients with cardiovascular diseases should receive corresponding treatment to attenuate the conditions, which may be beneficial to the reduction in the COVID-19 infection.

Lei et al. [53] combined the extracellular domain of human ACE2 with the Fc segment of human immunoglobulin IgG1 to generate the recombinant protein. In this study, they also employed fusion proteins from ACE2 mutants with low catalytic activity, and then, they featured the fusion proteins. Both fusion proteins showed high affinity for SARS-COV and SARS-COV-2 receptor-binding domains and were well validated in mice. In addition, the fusion protein balanced the pseudotype virus S protein of SARS-COV or SARS-CoV-2 in vitro. These fusion proteins show latent efficacy in the aspect of diagnosis, prevention, and treatment of SARS-COV-2, because of their crossreaction to coronavirus.

Monteil et al. [43] reported that a clinical grade of hrsACE2 reduced SARS-CoV-2 recovery from Vero cells by a factor of 1,000–5,000. However, there was no such effect in an equivalent mouse rsACE2. Meanwhile, SARS-CoV-2 could directly infect engineered human blood vessel organoids and human kidney organoids [43]. Such progress was inhibited by hrsACE2. These data demonstrated that hrsACE2 could significantly block early stages of SARS-CoV-2 infections (Figure 1).

In summary, COVID-19 is a serious public health hazard. The primary therapeutic principle is to prevent the complications based on the symptomatic treatment. In addition, the underlying diseases were treated, in order to prevent secondary infection. Meanwhile, immediate treatment was given to support the organ function. Indeed, ACE2 and the inflammatory system played crucial roles in the ALI and ARDS. These results suggested that ACE2-based agents may be a promising candidate for treating COVID-19. In the future, further studies are required to investigate the efficacy of recombinant ACE2 protein or AT1 receptor blockers on lung diseases.

## Data Availability

All the data were available upon appropriate request.
